# Identification of extracellular matrix proteins secreted by human dermal fibroblasts cultured in 3D electrospun scaffolds

**DOI:** 10.1038/s41598-021-85742-0

**Published:** 2021-03-23

**Authors:** Atena Malakpour-Permlid, Irina Buzzi, Cecilia Hegardt, Fredrik Johansson, Stina Oredsson

**Affiliations:** 1 Department of Biology, Lund University, Solvegatan 35C, 223 62 Lund, Sweden; 2 Division of Oncology, Department of Clinical Sciences Lund, Lund University, 223 81 Lund, Sweden

**Keywords:** Cancer, Breast cancer, Cancer models

## Abstract

The appreciation that cell interactions in tissues is dependent on their three dimensional (3D) distribution has stimulated the development of 3D cell culture models. We constructed an artificial 3D tumour by culturing human breast cancer JIMT-1 cells and human dermal fibroblasts (HDFs) in a 3D network of electrospun polycaprolactone fibres. Here, we investigate ECM components produced by the cells in the artificial 3D tumour, which is an important step in validating the model. Immunostaining and confocal fluorescence microscopy show that the ECM proteins fibronectin, collagen I, and laminin are deposited throughout the entire 3D structure. Secreted soluble factors including matrix metalloproteinases (MMPs) and interleukine-6 (IL-6) were analysed in collected medium and were found to be mainly derived from the HDFs. Treatment with transforming growth factor-β1 (TGF-β1), a major cytokine found in a tumour, significantly alters the MMP activity and IL-6 concentration. In addition, TGF-β1 treatment, changes the morphology of the HDFs to become more elongated and with increased linearized actin filaments compared to non-treated HDFs. Collectively, these novel findings suggest that the artificial 3D tumour displays a clear cell distribution and ECM deposition that resembles a tumour environment in vivo, suggesting an innovative biological model to study a human tumour.

## Introduction

The extracellular matrix (ECM) is the non-cellular network of various structural and adhesive macromolecules that are essential for many fundamental cellular processes^1^. It is mainly composed of fibrous, insoluble, and high molecular weight proteins such as collagens and fibronectin that provide physical and mechanical support for cells and also activate many cellular and intracellular signalling pathways^2–4^. The other main class of structural macromolecular ECM components are the proteoglycans that fill the majority of the extracellular interstitial space within the tissue in the form of a hydrated gel. Furthermore, the ECM is composed of globular and soluble proteins *e.g.* growth factors and cytokines that are associated with structural proteins that direct cellular recruitment, elicit signal transduction, and regulate gene transcription^2,4,5^. The three-dimensional (3D) ECM has complex multifactorial roles supplying stability and support for the cells and is also involved in and regulating the myriads of signals that influence cell fate. In the tumour microenvironment (TME), the ECM is markedly deranged compared to the ECM in normal tissue and exerts a strong influence on tumour initiation and development^2,6^.

Fibroblasts are intimately linked to the ECM and to a large extent, they are responsible for the synthesis of the fibrillar constituents of the stroma. In addition, they regulate the function of adjacent epithelial cells through bidirectional interaction and secretion of growth factors and cytokines^7^. Contrary to the highly organized normal tissue ECM, the tumour ECM is associated with significant architectural changes characterized by unbalanced deposition and modified structure of the ECM proteins resulting in increased matrix stiffness and increased ECM volume^2,7,8^. Activated fibroblasts are responsible for the malignant ECM remodelling, which supports cancer cell metastasis and invasion^9,10^. The major proteolytic enzymes involved in ECM remodelling are the matrix metalloproteinases (MMPs)^11^. It has been reported that increased collagen deposition and cross-linking promotes tumour initiation and malignant behaviour^12^. During tumour progression, stiffened ECM stimulates fibroblasts proliferation and activity further through mechanically-driven secretion of transforming growth factor-β1 (TGF-β1)^13^. TGF-β1 is a multifunctional cytokine that has been shown to promote fibroblast proliferation and drive the stable accumulation of ECM proteins particularly collagen and fibronectin in vivo^14,15^. In addition, it is well-established that TGF-β signalling is involved in epithelial to mesenchymal transition (EMT) during cancer progression contributing to metastasis^16^. It has been reported that TGF-β1 and other cytokines secreted by cancer cells and stromal cells can induce the transition of a normal fibroblast into a cancer associated fibroblast (CAF)^17–19^. Another pro-tumorigenic cytokine found in the TME is interleukin-6 (IL-6), which can be secreted by both fibroblasts and cancer cells^20–22^.

It is crucial that pre-clinical biological research can be performed under conditions that closely resemble the native microenvironment in vivo^23^. This has resulted in the development of 3D cell culture models that better mimic in vivo physiology compared to conventional two-dimensional cell culturing. 3D cell culturing allows the cells to interact with their surroundings in all three dimensions in a 3D environment^24^. Approaches to 3D culturing can be based on using 3D solid scaffold-based cultures, with scaffolds made from either natural or synthetic materials, or scaffold-free 3D models^25^.

Our group has previously described a custom-designed 3D fibrous scaffold made of polycaprolactone (PCL) resembling the collagen network of the ECM used for 3D cell culturing of normal cells and cancer cells^26^. We have shown that normal and cancer cells proliferate in the entire 3D scaffolds as mono- and co-cultures and they can be used as a miniaturized 3D tumour model for therapeutic studies (unpublished data). Here, we characterised the deposition of fibronectin, collagen I, and laminin in such 3D PCL-based mono-cultures of human dermal fibroblasts (HDFs) and co-cultures of JIMT-1 human breast cancer cells and HDFs incubated in the absence or presence of TGF-β1. We also determined the medium level of the IL-6 cytokine and the MMP activity under the different culture conditions. The presented results show a progressive increase in the deposition of major ECM components including fibronectin, collagen, and laminin and that the pattern of deposition changes during the incubation time, and this was affected by TGF-β1 treatment. We believe it is important to characterize the cell-derived ECM proteins in 3D cell culture models to be able to use them optimally for biological studies of a tumour in vitro.

## Materials and methods

### Preparation of 3D scaffolds

The highly porous custom-designed 3D PCL fibre scaffolds were purchased from Cellevate AB (Lund, Sweden). The randomly-oriented PCL fibre meshes were subjected to O_2_ plasma treatment for 10 s in order to increase the surface hydrophilicity and cell attachment^26^. Then, the scaffolds were sterilized with 99.5% ethanol for 15 min and were rinsed three times with sterile phosphate-buffered saline (PBS) prior to cell seeding.

### Cell lines and conditions

The human breast carcinoma cell line JIMT-1 (ACC-589) was purchased from the German Collection of Microorganisms and Cell Cultures (Braunschweig, Germany). The cells were tested for mycoplasma during the experimental period and were found to be negative (Eurofins Scientific, Cologne, Germany). HDFs (106-05a) were purchased from Sigma-Aldrich Sweden AB (Stockholm, Sweden). The HDFs were maximally used between passages 3–6. The cell lines were cultured in Dulbecco’s modified Eagle medium/Ham´s F-12 (1:1) supplemented with 5% heat-inactivated donor herd horse serum (DHHS) (Sigma-Aldrich Sweden AB), 5 μg/ml insulin (Sigma-Aldrich Sweden AB), 10 ng/ml epidermal growth factor (Lonza, Basel, Switzerland), 0.5 µg/ml hydrocortisone (Sigma-Aldrich Sweden AB), 1 mM Na-pyruvate (Sigma-Aldrich Sweden AB), 50 µg/ml transferrin (Sigma-Aldrich Sweden AB), 2 mM L-glutamine (VWR, Lund, Sweden), 1 mM non-essential amino acids (VWR), 100 μg/ml streptomycin (VWR), and 100 U/ml penicillin (VWR). The cell lines were maintained in a humidified incubator (95% humidity) with 5% CO_2_ in air at 37 ºC (CO_2_ incubator). The cells were passaged twice a week using Accutase (Sigma-Aldrich Sweden AB).

### 3D cell culture systems

The JIMT-1 cells and HDFs were detached from the culture flask using Accutase and the cell number determined by counting in a hemocytometer. Cell suspensions containing a defined number of JIMT-1 cells (15,000 cells/ml), HDFs (30,000 cells/ml), or JIMT-1 cells and HDFs (15,000 and 30,000 cells/ml, respectively) were prepared after detachment. Cell suspension (1 ml) was added to the PCL scaffolds placed in hydrophobic 48 well plates to form mono-cultures of JIMT-1 cells or HDFs and co-cultures of JIMT-1 cells and HDFs. The plates were kept in the incubator at 37 ºC for two weeks and the medium was renewed every 72 h of incubation.

### Transforming growth factor-β1 treatment

TGF-β1 was purchased from R&D Systems, Inc. (Minneapolis, MN, USA). A 10 μg/ml stock solution was prepared in sterile 4 mM HCl containing 0.1% human (P6140, Biowest, Nuaillé, France) or bovine serum albumin (A7906, Sigma-Aldrich Sweden AB). The stock solution was kept at −20 °C. When used, TGF-β1 was added to a final concentration of 5 ng/ml 24 h after cell seeding. Cell culture medium was collected (see below) after every 72 h of incubation and 1 ml fresh medium without or with 5 ng/ml TGF-β1 was added to each well.

### Collection of cell culture medium and cell extracts

At the time of medium change on days 3, 7, 10, and 14 after seeding, the old cell culture medium was collected for IL-6 analysis. The medium from each well was transferred into sterile Eppendorf tubes. Then, collected medium was clarified by centrifugation for 10 min at 1000 g and the supernatants were kept frozen at −20 °C for further analysis.

### Decellularization and fixation

Following medium collection on days 3, 7, 10, and 14 after seeding, the 3D scaffolds were washed three times with sterile PBS. Then, 1 ml of pre-warmed (37 °C) extraction buffer (1% Tween-20 and 20 mM NH_4_OH in PBS) was added to the wells with washed scaffolds and they were incubated at 37 °C for 5 min. After decellularization, the 3D cultures were rinsed three times with sterile PBS and fixed in 3.7% formaldehyde in PBS for 15 min at 4º C. Then, the scaffolds were washed three times with PBS and kept at 4 ºC in 25% ethanol in PBS until immunofluorescence staining was performed. In addition, some cultures were fixed as described above without decellularization.

### Immunofluorescence staining

The ECM of the decellularized samples was stained for visualization of laminin, fibronectin, or collagen I separately. For ECM staining, the fixed samples were incubated in blocking buffer (1% bovine serum albumin and 1% Tween 20 in PBS) at room temperature for 1 h. Then, the cultures were incubated with anti-fibronectin polyclonal primary antibody (ab2413, 1:200) (Abcam, Cambridge, United Kingdom), anti-laminin polyclonal primary antibody (PA5-16287, 1:250) (ThermoFisher Scientific, Carlsbad, CA, USA), or anti-collagen I polyclonal primary antibody (PA1-26204, 1:200) (ThermoFisher Scientific) for 2 h at room temperature. The cells were washed 3 times with PBS and incubated 2 h at room temperature with the secondary antibody Alexa Flour 488 anti-rabbit (A11034, 1:800) (ThermoFisher Scientific). Cultures with cells were labelled for detection of fibronectin as described above and then the cytoskeleton was stained with Alexa Flour 594 phalloidin (A12381, 1:100) (ThermoFisher Scientific). Then, the cultures were washed three times with PBS and the cell nuclei were stained with 4′,6-diamidino-2-phenylindole DAPI (1 µg/ml) (ThermoFisher Scientific) for 2 min at room temperature. All samples were stored in PBS protected from light before microscopy.

### Confocal laser scanning microscopy

For visualization of cell and ECM distribution in 3D scaffolds, stained samples were mounted with RapiClear 1.49 clearing agent (10 μl) (SunJin Lab Co., Hsinchu, Taiwan). Images were taken with a Leica SP8 DLS inverted confocal laser scanning microscope and oil immersion 20 × objective lenses (Leica Microsystems, Wetzlar, Germany).

### Fluorescent intensity quantifications

Images of fluorescently labelled samples for detection of laminin, fibronectin, and collagen I were obtained with an Olympus/Nikon AX70 epifluorescence microscope (20 × objective lens with numerical aperture 0.50) (Olympus Optical Co. Ltd., Japan) equipped with a digital camera (Nikon Imaging Japan Inc.). The same acquisition settings were used for all the images taken for each protein. Three independent cultures were imaged for each experimental set up and three images were taken per culture. Measurements of total fluorescence intensity of the proteins were determined using the Fiji ImageJ software version 1.52r (http://imagej.nih.gov/ij/). The mean total fluorescence intensity of the three images was taken as the fluorescence intensity for each independent culture. Then, the mean of the three fluorescence intensities for the same culture condition was calculated ± SEM.

### Cryosectioning of 3D scaffolds

After microscopy, the stained 3D cultures were embedded in tissue freezing medium Optimal Cutting Temperature Cryomount (Histolab Products AB, Västra Frölunda, Sweden) for 30 min at room temperature and they were kept at −20 °C until cryosectioning. Cryosectioning was performed using a Leica CM1950 Cryostat (Leica Biosystems, Nüssloch, Germany) at −20 °C. The samples were first trimmed and then sections of samples were cut perpendicular to the surface of the filter thus giving a view of the depth. The sections were collected on Polysine glass slides (Manzel GmbH and Co., Bielefeld, Germany) afterwards. The samples were washed three times with PBS to remove the freezing medium. They were mounted with Mowiol (Sigma-Aldrich Sweden AB) and stored at −20 °C until microscopic visualization. The samples were protected from light during the microscopy imaging. Images were acquired using an Olympus AX70 (Olympus Optical Co. Ltd., Japan) fluorescence microscope equipped with a digital camera (Nikon Imaging Japan Inc, Tokyo, Japan).

### Enzyme-linked immunosorbent assay (ELISA)

The collected medium was thawed on ice and centrifuged for 10 min at 1000 g. IL-6 in the culture medium was quantified using an IL-6 Human ELISA Kit (BMS213-2, ThermoFisher Scientific) according to the manufacturer's instructions. The enzyme-mediated transformation of the chromogen into a coloured product was assayed measuring the 450 nm absorbance of each well using a SpectraMax i3x multi-mode microplate reader (Molecular Devices, San Jose, CA, USA). The intensity of the coloured product is directly proportional to the concentration of antigen present in the sample.

### Analysis of matrix metalloproteinase activity

The MMP activity in the medium from cultures incubated for 14 days was determined using a MMP Activity Assay Kit (ab112147, Abcam). The kit is designed to check the general activity of MMP enzyme activity. The fluorescence intensity was determined using a SpectraMax i3x multi-mode microplate reader set at Ex/Em 540/590 nm.

### Statistical analysis

Results are expressed as the mean ± SEM. The paired Student’s t-test was used for statistical analysis. Differences were considered statistically significant when *p* < 0.05.

## Results

### ECM protein deposition in the 3D cell cultures

Here, we investigate the cellular deposition of the ECM proteins, fibronectin, collagen I, and laminin in 3D PCL-based scaffold cultures of HDFs or HDFs co-cultured with JIMT-1 breast cancer cells. Immunostaining was used to detect the proteins and their distribution in decellularized 3D scaffolds after 3, 7, 10, and 14 days of cell cultures incubated in the absence or presence of TGF-β1. Overall, deposition of the different ECM proteins was observed to increase with longer incubation time.

**Figure 1:**
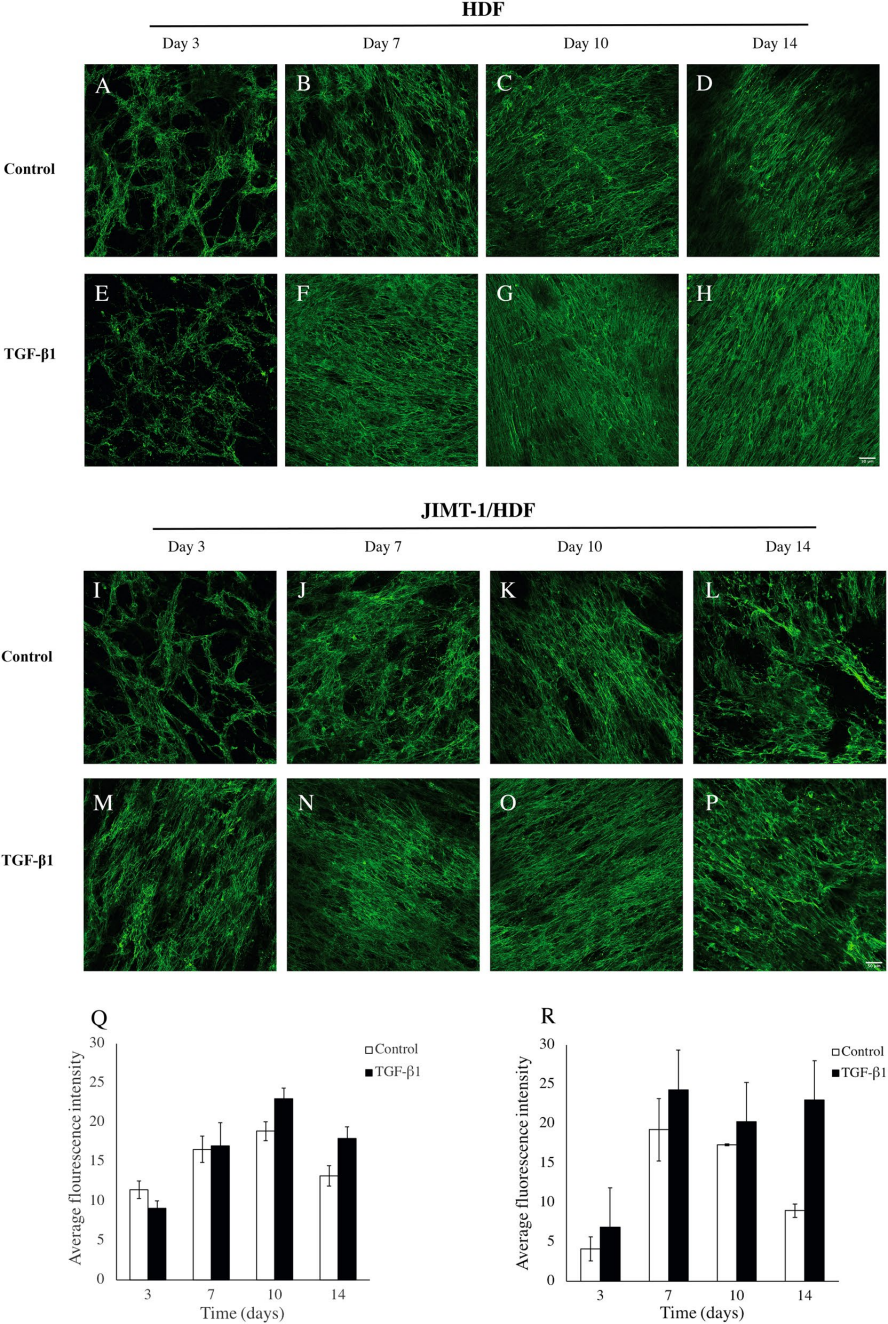
Fibronectin deposition, localization, and quantification in decellularized scaffolds of HDF and JIMT-1/HDF 3D cultures. (**A**–**P**) Single confocal plane images of fibronectin deposition taken in the centre of decellularized 3D cultures incubated in the absence (control) or presence of 5 ng/ml TGF-β1. The 3D cultures were decellularized, fixed in 3.7% formaldehyde and stained to visualize fibronectin (green) at 3, 7, 10, and 14 days of culturing. All images are representative of 3 independent experiments. Scale bar is 50 μm. Quantification of fluorescence intensity using ImageJ in images taken by epifluorescence microscopy in HDF decellularized scaffolds (**Q**) and JIMT-1/HDF decellularized scaffolds (**R**). The columns are represented as mean ± SEM (n = 3).

The pattern of fibronectin deposition in 3D HDF mono-cultures was netlike on day 3 and then the net like structure became denser on days 7 to 14 on the 3D PCL-based scaffold (Fig. 1A–H). TGF-β1 treatment appeared to result in a somewhat denser fibronectin deposition network at least at day 14 after seeding (compare Fig. 1D, H). In the JIMT-1/HDF co-cultures, the fibronectin deposition was not as evenly distributed as in the 3D HDF mono-cultures. There are less dense areas in the pattern (Fig. 1 I–P), and as seen below (Fig. 6), these are areas where the cancer cells were located before decellularization. The effect of TGF-β1 treatment on the fibronectin deposition pattern in the JIMT-1/HDF co-culture was similar to that in the HDF 3D mono-culture.

We attempted to get an estimation of the amount of deposited fibronectin by measuring the fluorescence intensities applying the software Image J to images taken by epifluorescence microscopy and the data are shown in Fig. 1 Q and R. This method only gives a rough estimation of the relative changes in the fibronectin content during the 14 incubation days. The fluorescence signals of the HDF mono-culture and the JIMT-1/HDF co-culture cannot be directly compared, however, it is possible to compare control and TGF-β1 within each culture condition. In the HDF 3D cultures, the fibronectin fluorescence intensity increased from day 3 to day 10 with no significant difference between control and TGF-β1-treated cultures (Fig. 1Q) and then there was a decrease on day 14. In the 3D co-culture, the fibronectin content increased between days 3 and 7 and then stayed at a plateau in TGF-β1-treated cultures (Fig. 1R). In the control, there was a marked decrease in fibronectin between day 10 and 14. These quantification data are to some degree reflected in the images obtained by confocal microscopy. However, the images only show one representative confocal plane in the centre of the 3D scaffold.

**Figure 2:**
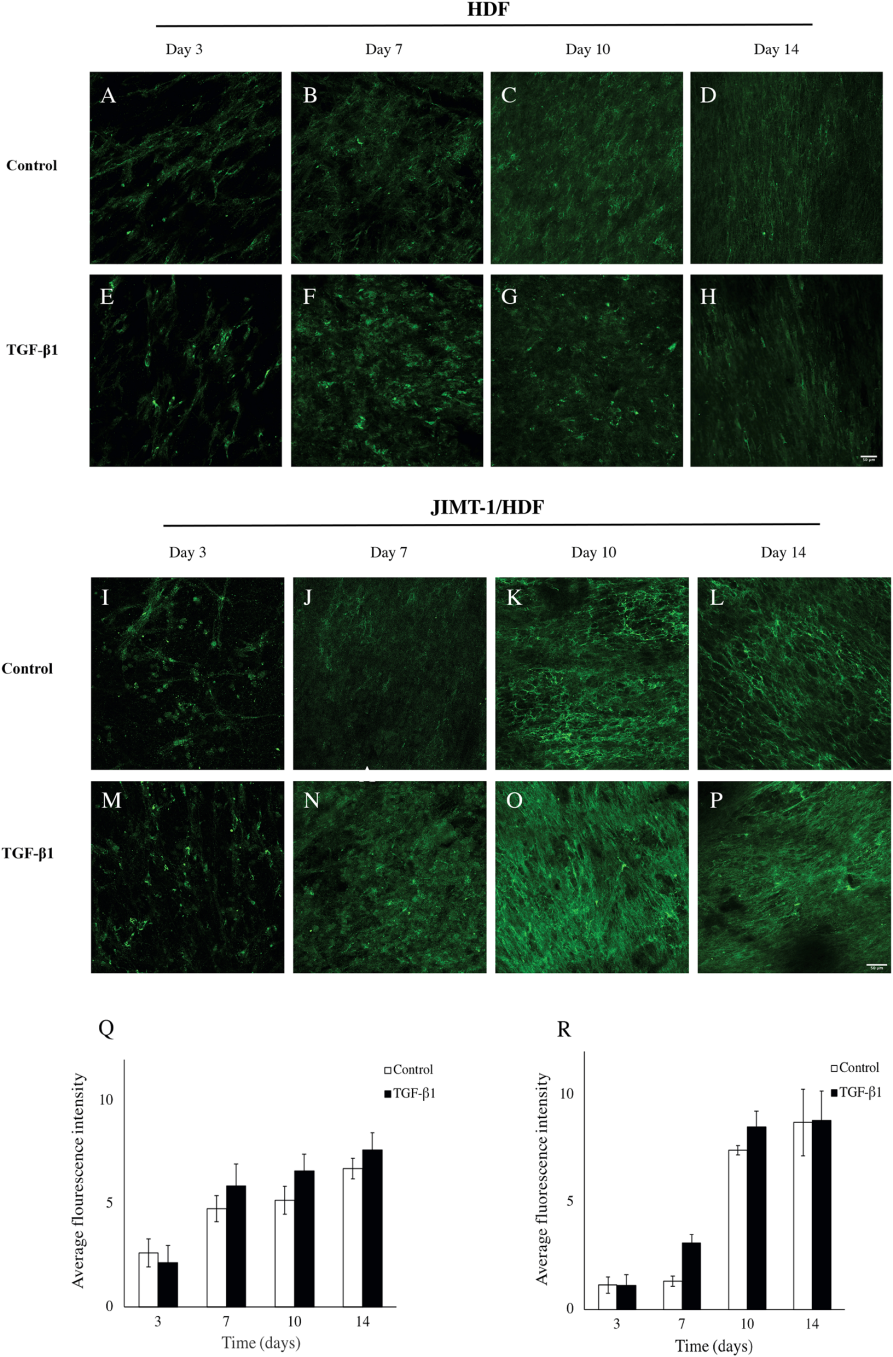
Collagen I deposition, localization, and quantification in decellularized scaffolds of HDF and JIMT-1/HDF 3D cultures. (**A**–**P**) Single confocal plane images of collagen I deposition taken in the centre of decellularized 3D cultures incubated in the absence (control) or presence of 5 ng/ml TGF-β1. The 3D cultures were decellularized, fixed in 3.7% formaldehyde and stained to visualize fibronectin (green) at 3, 7, 10, and 14 days of culturing. All images are representative of 3 independent experiments. Scale bar is 50 μm. Quantification of fluorescence intensity using ImageJ in images taken by epifluorescence microscopy in HDF decellularized scaffolds (**Q**) and JIMT-1/HDF decellularized scaffolds (**R**). The columns are represented as mean ± SEM (n = 3).

TGF-β1 treatment did not appear to affect the deposition of collagen in 3D HDF mono-cultures (Fig. 2A–H). Neither do the images of TGF-β1 treatment of co-cultures show any difference in collagen I deposition compared to control co-cultures (Fig. 2 I–P). Comparing the confocal images taken of HDF 3D mono-cultures days 10 and 14 with those taken of JIMT-1/HDF co-cultures the same days, gives the impression that there was more collagen I in the co-cultures (compare Fig. 2C, D, G, and H with K, L, O, and P).

Evaluation of the fluorescence intensities in images of collagen I deposition taken by epifluorescence microscopy as described above, revealed increased collagen I level during days 3 to 14 in HDF 3D mono-cultures (Fig. 2Q). In the co-culture, collagen I increased markedly between days 7 and 10 with the same pattern in control as in TGF-β1-treated cultures (Fig. 2R).

**Figure 3:**
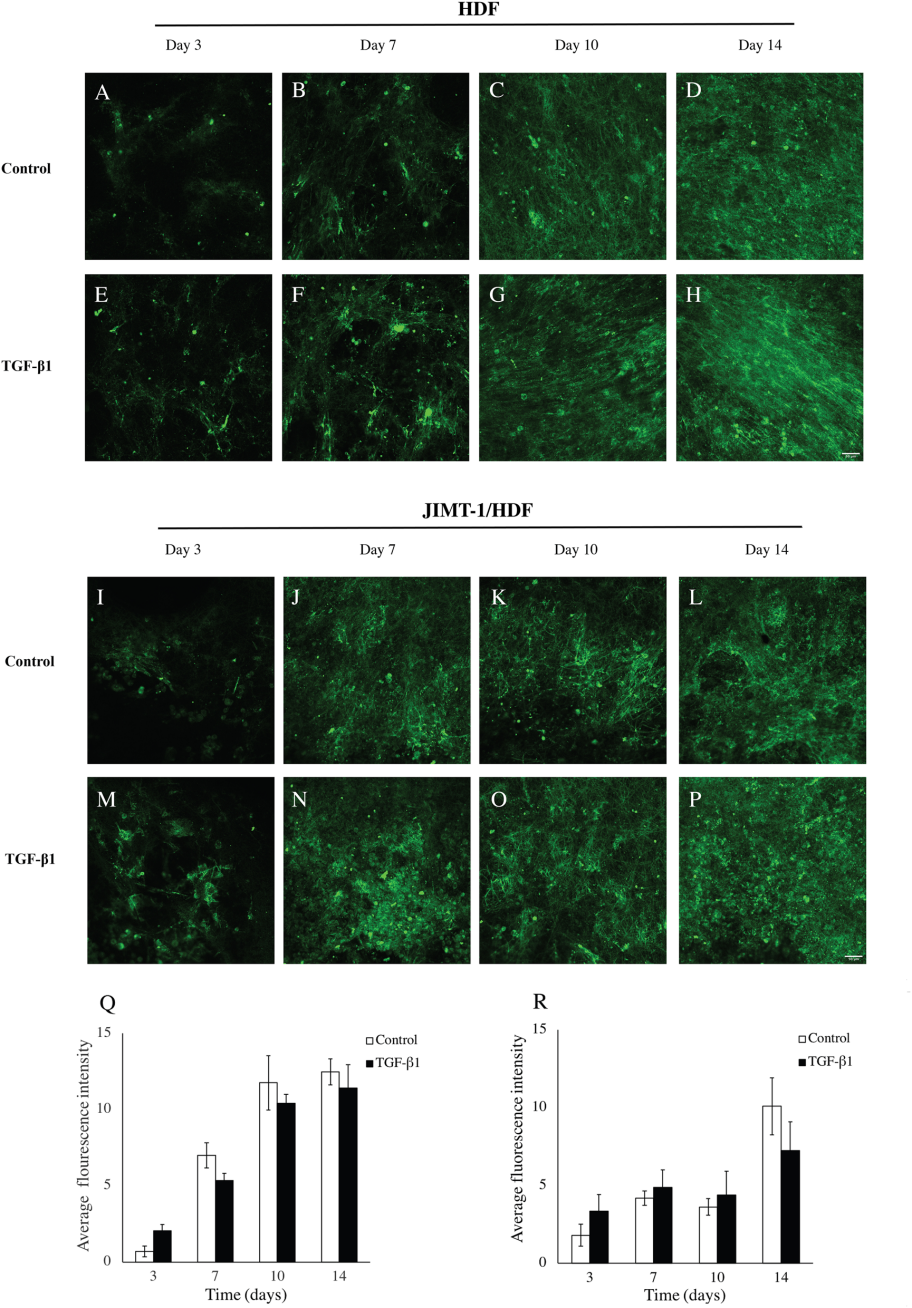
Laminin deposition, localization, and quantification in decellularized scaffolds of HDF and JIMT-1/HDF 3D cultures. (**A**–**P**) Single confocal plane images of laminin deposition taken in the centre of decellularized 3D cultures incubated in the absence (control) or presence of 5 ng/ml TGF-β1. The 3D cultures were decellularized, fixed in 3.7% formaldehyde and stained to visualize fibronectin (green) at 3, 7, 10, and 1 days of culturing. All images are representative of 3 independent experiments. Scale bar is 50 μm. Quantification of fluorescence intensity using ImageJ in images taken by epifluorescence microscopy in HDF decellularized scaffolds (**Q**) and JIMT-1/HDF decellularized scaffolds (**R**). The columns are represented as mean ± SEM (n = 3).

The amount of laminin deposited was low on day 3 of incubation and increased during the experimental period and the level of laminin was similar in the two treatment conditions (absence Fig. 3A–D or presence Fig. 3E–H of TGF-β1). Confocal images of the co-cultures, show that the laminin deposition is similar as the deposition in the HDF mono-culture (Fig. 3I–P).

Evaluation of the fluorescence intensities in images taken by epifluorescence microscopy as described above, revealed increased laminin level during days 3 to 14 with only a trend to less laminin in cultures treated with TGF-β1 compared to cultures incubated in the absence of TGF-β1 (Fig. 3Q). In the co-culture, laminin deposition as evaluated by the fluorescence intensities in images taken by epifluorescence microscopy was similar in control and TGF-β1-treated cultures with a trend to decreased deposition day 14 (Fig. 2R).

### Fibronectin localization in the 3D cell cultures

**Figure 4:**
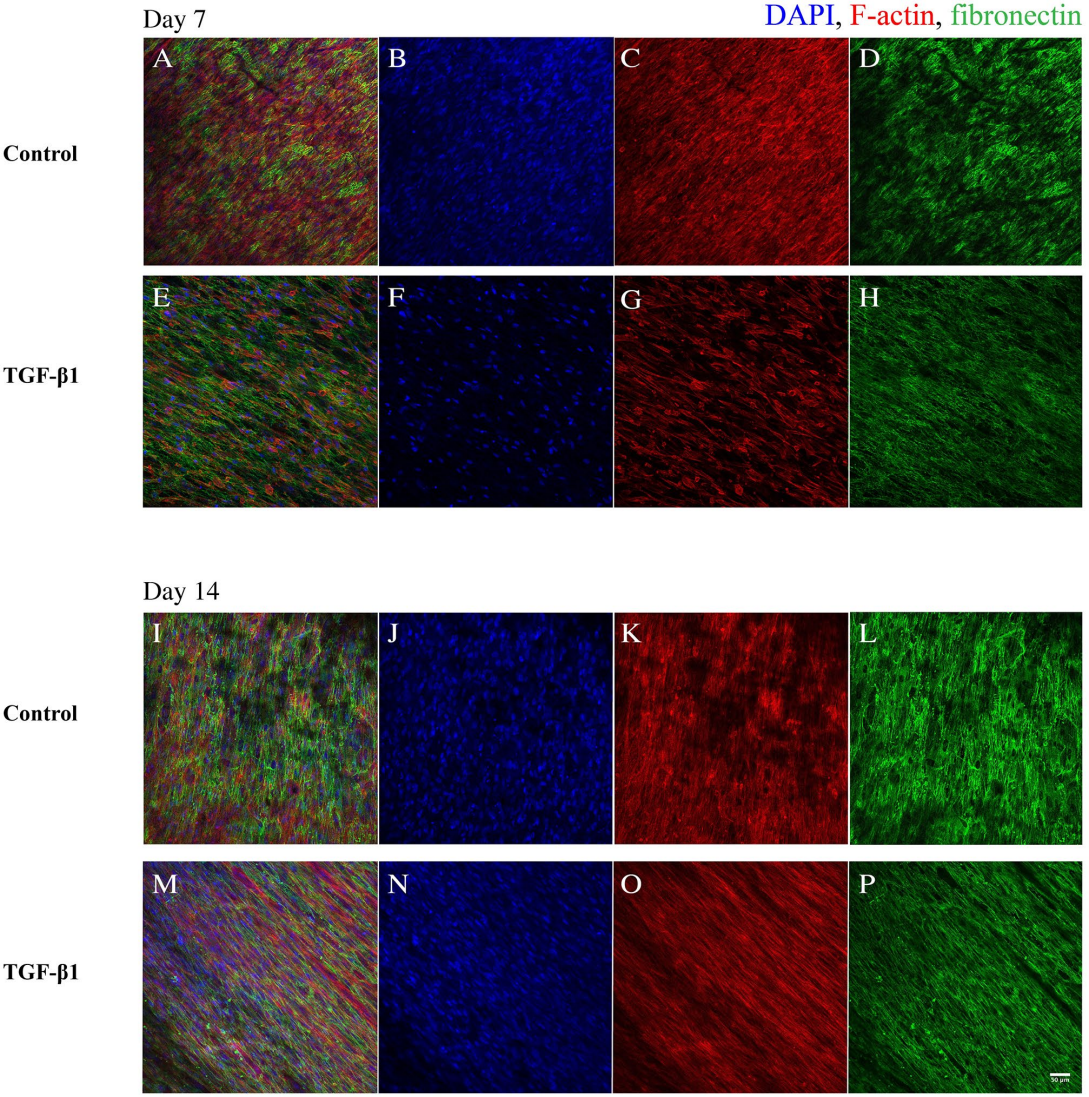
Confocal images of fibronectin co-localization in HDF 3D mono-cultures in response to TGF-β1 treatment. HDF 3D mono-cultures were incubated in the absence (control) or presence of 5 ng/ml TGF-β1 for 7 and 14 days in 3D scaffolds. Cultures were fixed in 3.7% formaldehyde and stained to visualize actin filaments (red), fibronectin (green) and, cell nuclei (blue) after 7 and 14 days in culture. The images were taken in the centre of 3D cultures. All images are representative of 3 independent experiments. Scale bar is 50 μm.

We have previously shown that HDFs grow in tight parallel sheets following the PCL fibre structure^26^. Here, we show that the fibronectin is deposited in alignment with the fibroblasts (visualized by actin staining) on the PCL fibre network (Fig. 4). In TGF-β1-treated cultures, the fibroblasts were more elongated and showed a denser linearized growth pattern compared to that of fibroblasts in cultures incubated without TGF-β1 which is specifically seen at 14 days of cultivation.

**Figure 5:**
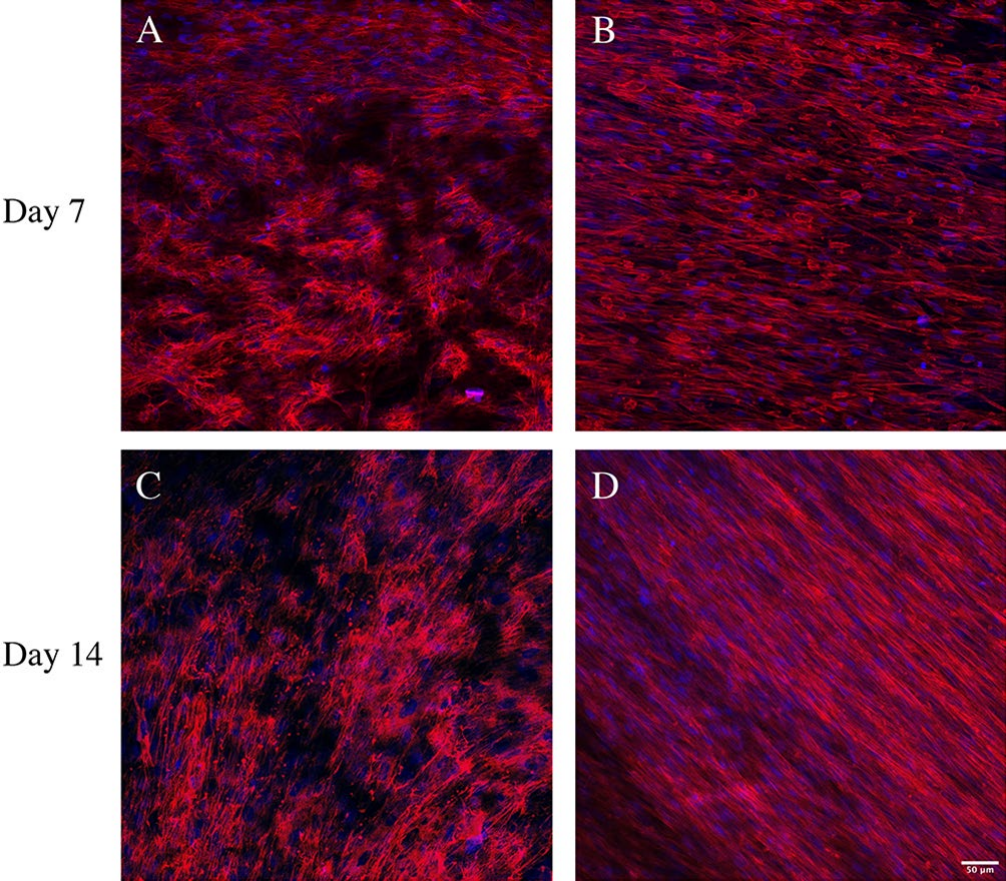
Confocal images of actin filaments in HDF 3D mono-cultures incubated in the absence or presence of TGF-β1. HDF 3D co-cultures were incubated in the absence (**A** and **C**) or presence of 5 ng/ml TGF-β1 (**B** and **D**) for 7 and 14 days in 3D scaffolds, respectively. The cultures were fixed in 3.7% formaldehyde and stained to visualize actin filaments (red) and cell nuclei (blue). The images were taken in the centre of 3D cultures All images are representative of 3 independent experiments. Scale bar is 50 μm.

Upon treatment with TGF-β1, there are changes in actin filament organization (Fig. 5). The actin filaments are short in control, while they become highly elongated and linearized in HDFs treated with TGF-β1.

**Figure 6:**
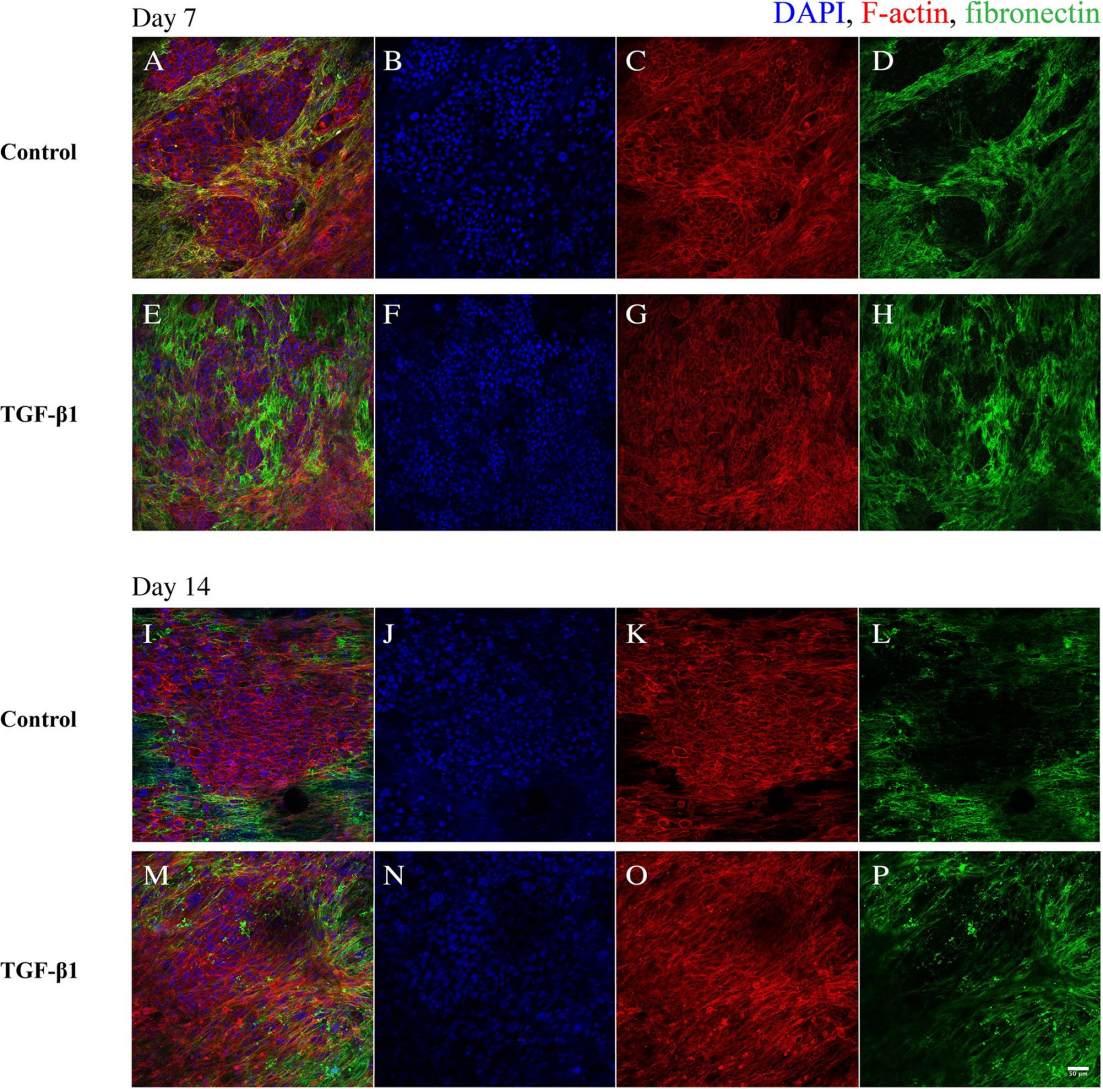
Confocal images of fibronectin localization in JIMT-1/HDF 3D co-cultures in response to TGF-β1 treatment. JIMT-1/HDF 3D co-cultures were incubated in the absence (control, **A**–**D** and **I**–**L**) or presence of 5 ng/ml TGF-β1 (**E**–**H** and **M**–**P**) for 7 and 14 days in 3D scaffolds. The co-cultures were fixed in 3.7% formaldehyde and stained to visualize actin filaments (red), fibronectin (green), and cell nuclei (blue) after 7 and 14 days in culture. The images were taken in the centre of 3D cultures. All images are representative of 3 independent experiments. Scale bar is 50 μm.

In the 3D co-cultures JIMT-1 cells and HDFs (Fig. 6), the JIMT-1 cells growing in tight clusters surrounded by streaks of fibroblasts which is similar to breast tumour histology^27^. Fibronectin is deposited in connection with the fibroblasts as the areas where cancer cells are located do not show fibronectin labelling (Fig. 6). In cultures incubated in the absence of TGF-β1, the JIMT-1 cells grow in tight clusters with some dispersed cells (Figs. 6A and I). TGF-β1 treatment seems to loosen up the JIMT-1 colonies which is apparent in Fig. 6 M. It seems like the cancer cells are spreading out along the streaks of elongated HDFs.

### 3D distribution of cells

**Figure 7:**
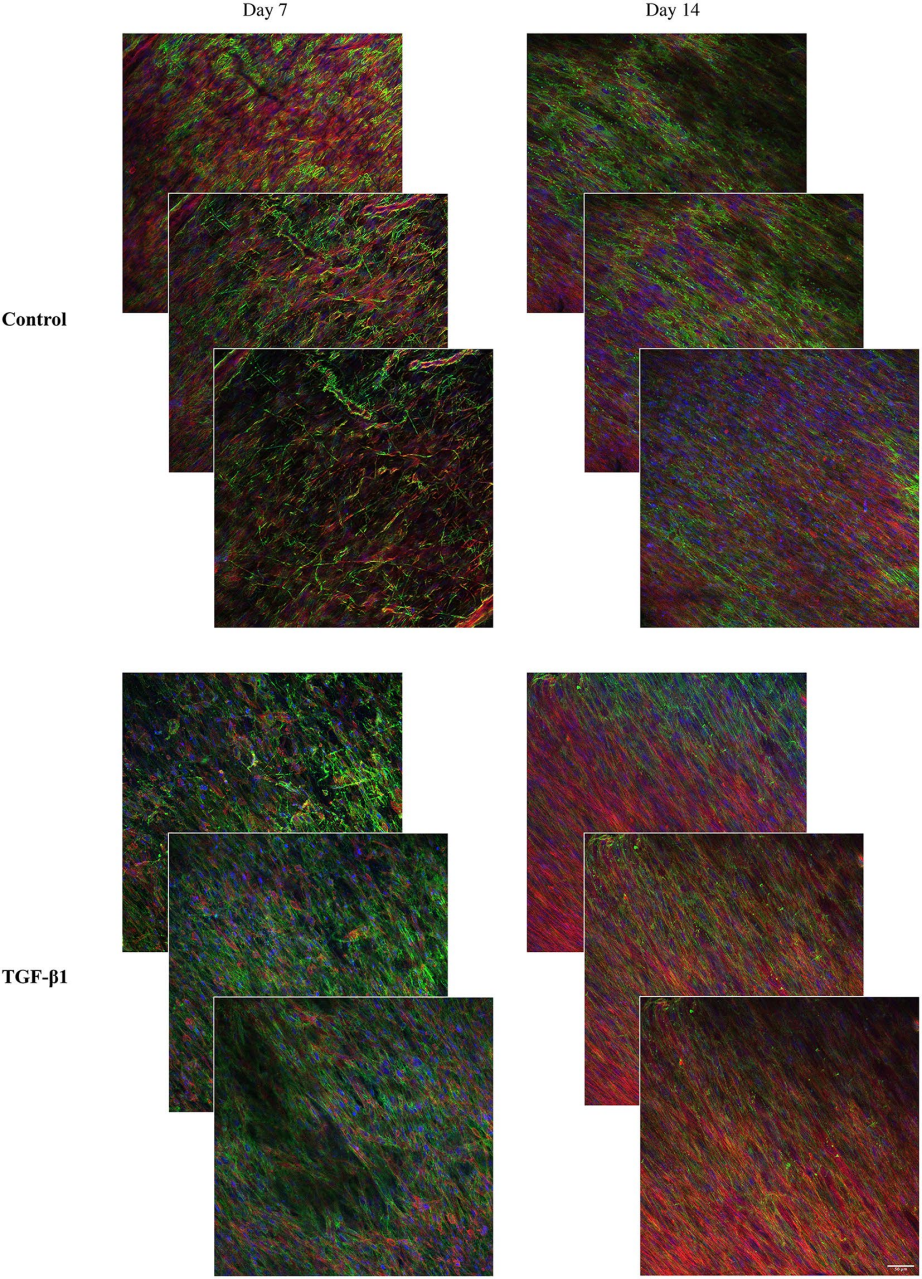
Focus stacking images obtained by confocal microscopy of fibronectin deposition and localization in 3D cultures of HDFs. Three confocal plane images were obtained with approximately 10 µm distance from each other in the 3D cultures. HDF 3D mono-cultures were incubated in the absence (control) or presence of 5 ng/ml TGF-β1 for 7 and 14 days. Cultures were fixed in 3.7% formaldehyde and labelled to visualize actin (red), fibronectin (green), and cell nuclei (blue). All images are representative of 3 independent experiments. Scale bar is 50 μm.

**Figure 8:**
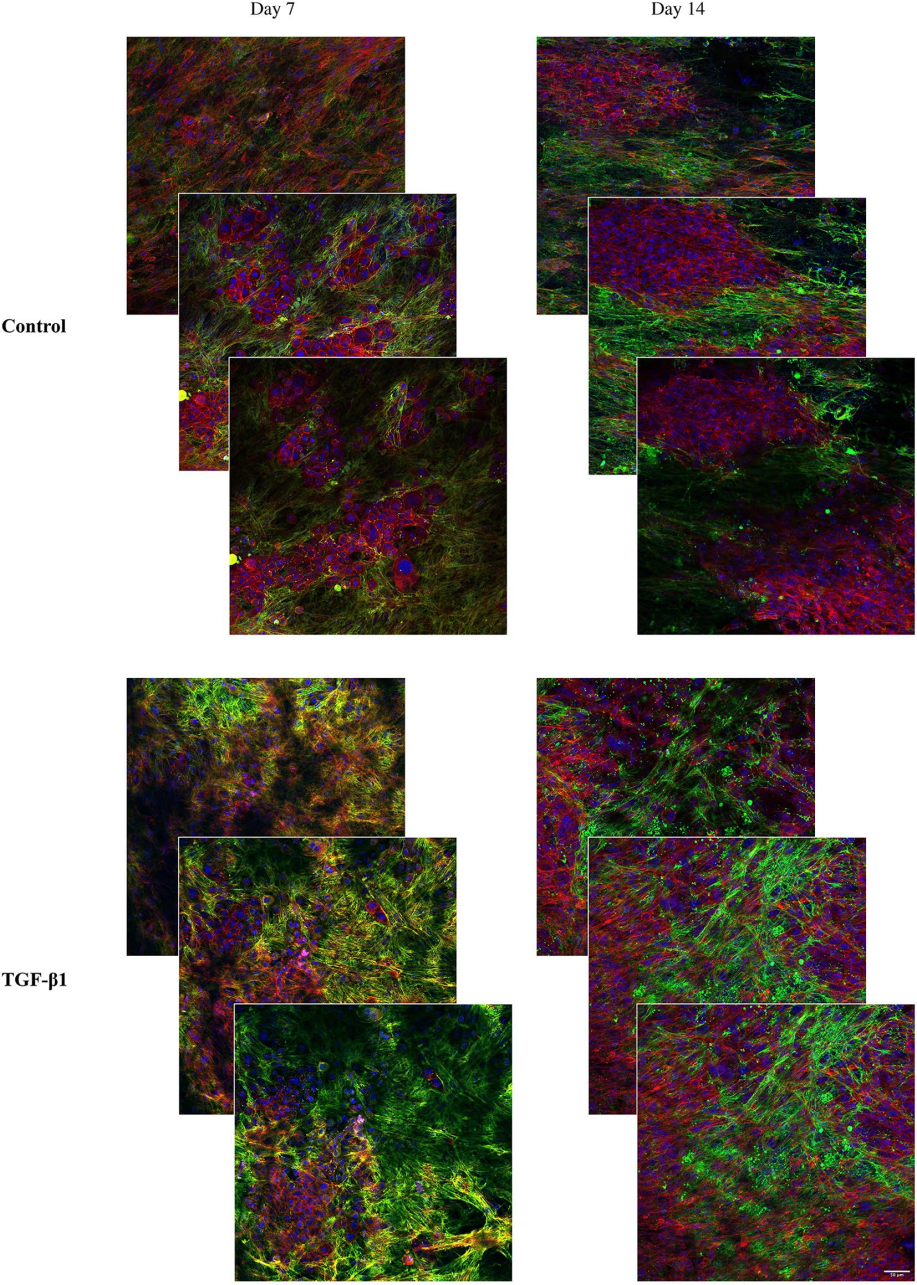
Focus stacking images obtained by confocal microscopy of fibronectin deposition and localization in 3D co-cultures of JIMT-1/HDFs. Three confocal plane images were obtained with approximately 10 µm distance from each other in the 3D cultures. JIMT-1/HDF 3D co-cultures were incubated in the absence (control) or presence of 5 ng/ml TGF-β1for 7 and 14 days. Cultures were fixed in 3.7% formaldehyde and labelled to visualize actin (red), fibronectin (green), and cell nuclei (blue). All images are representative of 3 independent experiments. Scale bar in 50 μm.

To give an impression of the 3D distribution of cells which is lost in the single plane confocal images shown above, three confocal planes in z direction of the 3D HDF mono-cultures and the 3D JIMT-1/HDF co-cultures are shown in Figs. 7 and 8, respectively. The approximate distance between the images is 10 µm with the middle image in the centre of the 3D scaffolds. The three confocal plane images of HDFs show the alignment of the HDFs and their tighter growth in cultures incubated with TGF-β1 compared to those incubated without TGF-β1 (Fig. 7). The three confocal plane images of co-cultures treated with TGF-β1 show the less dense JIMT-1 cell clusters compared to the dense clusters in the cultures incubated in the absence of TGF-β1 (Fig. 8). The localization of fibronectin in conjunction with the HDFs in the 3D JIMT-1/HDF co-cultures is also obvious in the three confocal planes.

**Figure 9:**
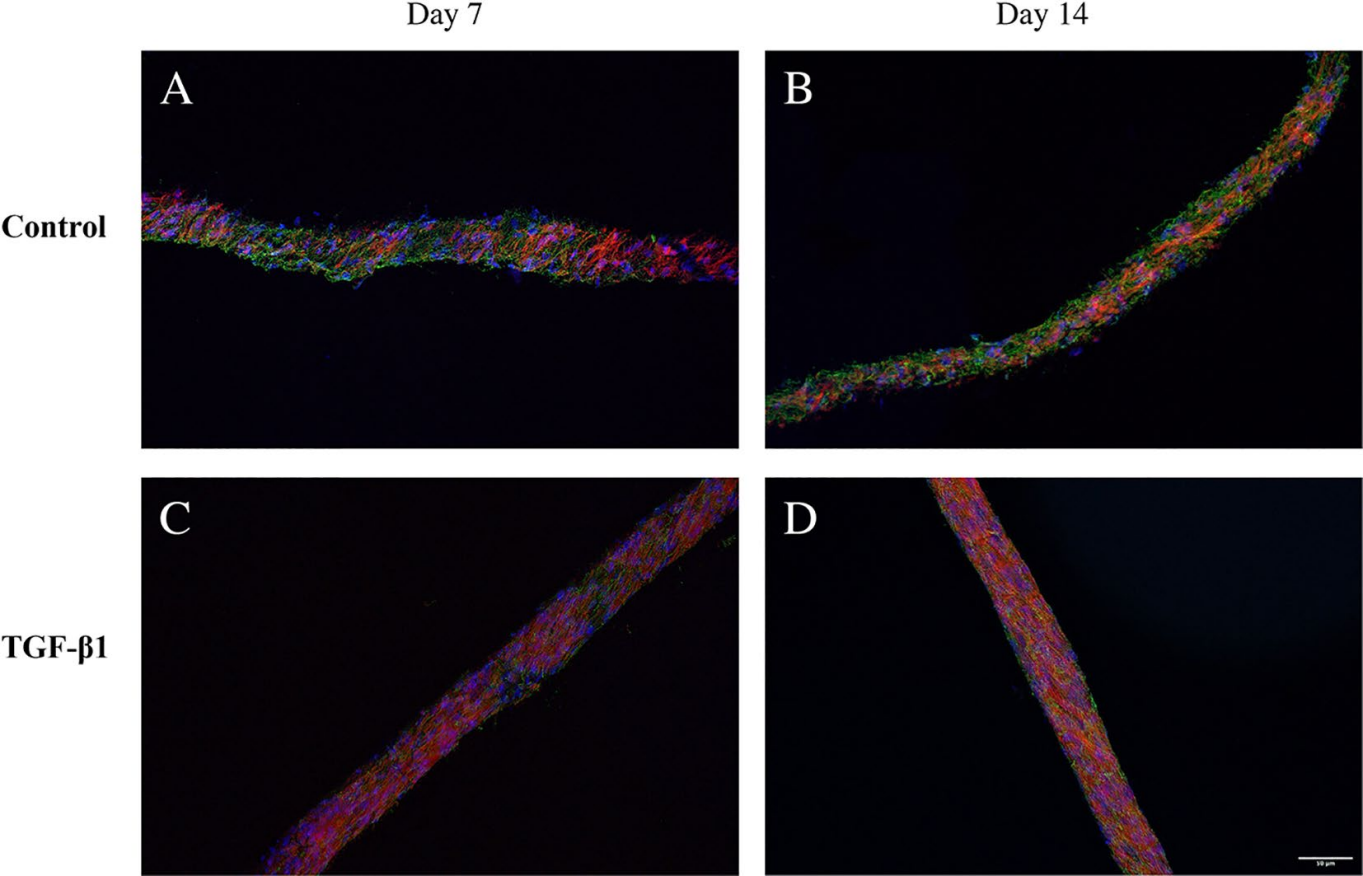
Fluorescence microscopy images of cryosectioned 3D mono-cultures of HDFs incubated in the absence or presence of TGF-β1. The HDF 3D cultures were incubated in the absence (control) or presence of TGF-β1 (5 ng/ml). After 7 and 14 days of incubation, cultures were fixed in 3.7% formaldehyde, stained to visualize actin filaments (red), fibronectin (green), and cell nuclei (blue), followed by cryosectioning, and fluorescence microscopy. All images are representative of 3 independent experiments. Scale bar is 50 μm.

**Figure 10:**
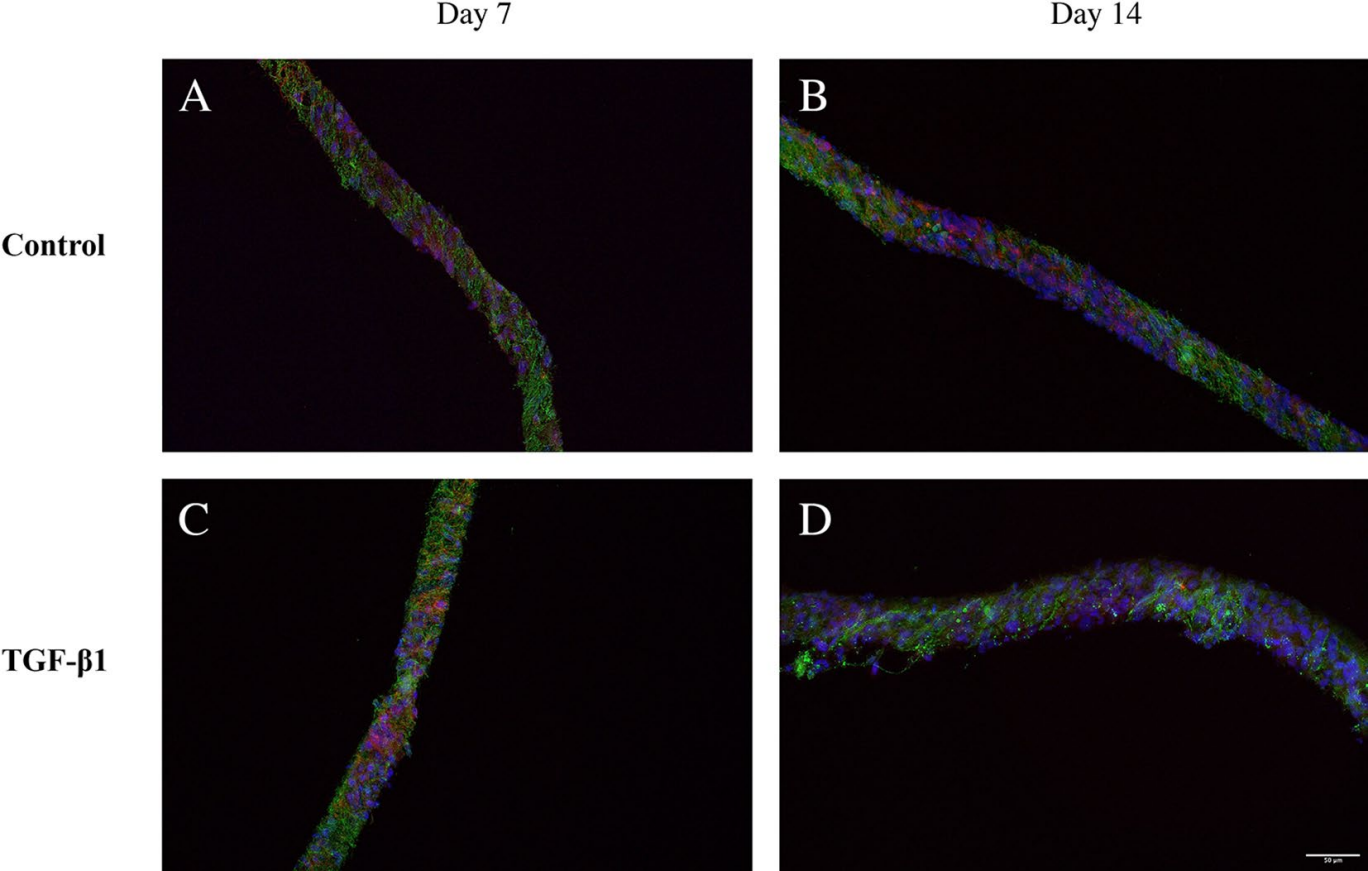
Fluorescence microscopy images of cryosectioned 3D JIMT-1/HDF co-cultures incubated in the absence or presence of TGF-β1. The JIMT-1/HDF 3D cultures were incubated in the absence (control) or presence of TGF-β1 (5 ng/ml). After 7 and 14 days of incubation, cultures were fixed in 3.7% formaldehyde, stained to visualize actin filaments (red), fibronectin (green), and cell nuclei (blue), followed by cryosectioning, and fluorescence microscopy. All images are representative of 3 independent experiments. Scale bar is 50 μm.

To fully assess cell growth and deposition of fibronectin in the entire depth of the scaffold, 40 µm thick cryosections of mono- and co-cultures of HDF and JIMT-1/HDF were obtained. Figures 9 and 10 show representative cross-sectional images of control and TGF-β1-treated HDF mono-cultures and JIMT-1/HDF co-cultures after 7 and 14 days of incubation, respectively. Cryosectioning confirm cell and fibronectin distribution in the entire depth of the PCL scaffolds.

### Cytokine secretion and MMP activity in the 3D cell culture

**Figure 11:**
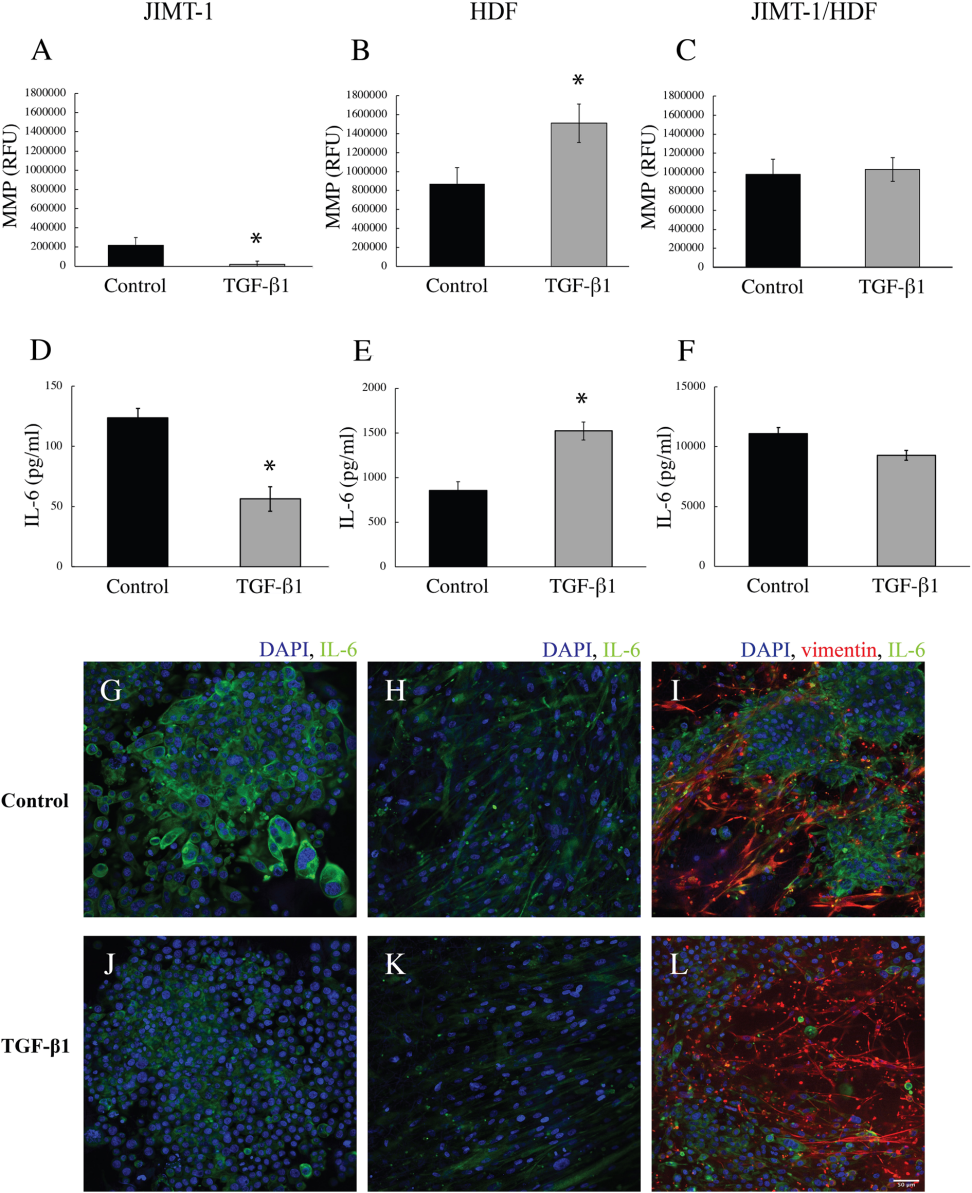
MMP activity and IL-6 level in the medium of JIMT-1 and HDF 3D mono-cultures and of JIMT1/HDF 3D co-cultures. The cultures were incubated in the absence (control) or presence of 5 ng/ml TGF-β1. After 14 days of incubation, the medium was collected for analysis of MMP activity (**A**–**C**) and the concentration of IL-6 (**D**–**E**). (**A**–**C**) The MMP activity was determined in 50 μl of medium by an assay based on the formation of a fluorescent product produced by MMP enzyme activity. (**D**–**F**) The level of IL-6 in the medium was determined by an ELISA assay. (**G**–**L**) Following the collection of the culture media, the cells were fixed with 3.7% formaldehyde and stained to visualize IL-6 (green), vimentin (red), and cell nuclei (blue). The images show a single confocal microscopy plane taken in the centre of the cultures. Each column represents mean ± SEM (n = 5). Students *t*-test, *P < 0.05. Scale bar is 50 μm.

The medium of JIMT-1 and HDF mono-cultures and of JIMT-1/HDF co-cultures was collected for analysis of the concentration of IL-6 and of the activity of MMP after 14 days of incubation (Fig. 11). The MMP activity was higher in the medium of HDFs compared to that in the medium of JIMT-1 cells (Fig. 11A and B). In JIMT-1 cells, TGF-β1 treatment significantly reduced the MMP activity while the MMP activity significantly increased in the medium of TGF-β1-treated HDFs (Fig. 11A and B). Medium of the JIMT-1/HDF co-cultures had a similar MMP activity as the medium of HDFs and TGF-β1 treatment did not affect the activity (Fig. 11C). The concentration of IL-6 in the medium followed the same pattern as the MMP activity (Fig. 11D–F). Noteworthy though is that the concentration of IL-6 differed markedly in the medium of the different cultures (note the different y-scales of Fig. 11D–F). The medium of JIMT-1 cells had approximately a sixfold lower concentration of IL-6 compared to the medium of HDFs, while the co-culture had approximately a 14-fold higher concentration of IL-6 in the medium compared to the medium of HDFs. Because of these differences, we decided to investigate the intracellular IL-6 by immunofluorescence with the thought that differences in intracellular expression may partly explain medium concentrations, a hypothesis that, however, is not supported by the images (Figs. 11G–L). IL-6 was found in both JIMT-1 cells and in HDFs and TGF-β1 treatment did not affect the intracellular IL-6. In Figs. 11I and L it is difficult to localise the IL-6 due to the strong vimentin staining in HDFs. Supplemental Figure S1 shows the different channels with similar expression of IL-6 in JIMT-1 cells and HDFs as shown in the mono-cultures.

## Discussion

The traditional perspectives of cancer biology have shifted from the study of mainly cancer cells to include the stromal cells as well as the ECM^28^. The ECM has a vital role in the reciprocal interactions between cancer cells and stromal cells in regulating tumour progression and migration. In many solid tumours, the ECM comprises up to 60% of the tumour mass and it is secreted to a large degree by fibroblasts^6^. There are numerous articles describing the ECM as well as articles describing 3D scaffolds that are supposed to mimic the ECM^29^. Despite this fact, there are not may studies that characterise the actual ECM formed in the context of artificial 3D scaffolds used in cell culturing. To this end, we decided to initiate a study of ECM proteins deposited in an in vitro 3D model that we had developed and used for evaluation of toxic compounds. The goal is to recreate a human tumour outside the body that can be used in pre-clinical studies of tumour development and to be used for efficient evaluation of anti-cancer compounds.

The electrospun 3D scaffolds used in this work consist of a 3D PCL fibre network, which represent the topography of collagen fibres in human tissues^26^. Our group has used this model system to investigate normal and cancer cell proliferation, cell morphology, cell distribution, and for pre-clinical cytotoxicity testing of different anti-cancer drugs^26^. The composition of the culture system is a combination of user-defined and cell-defined features. We provide the cells with a 3D artificial collagen structure mimicking scaffold as well as medium. The cells grow in the structure and form ECM. It should be noted that the medium contains DHHS and not fetal bovine serum in an effort to reduce the number of products surrounded with animal ethical concerns^30^. Obtaining blood samples from horses trained to be blood donors mainly for medicinal purposes cause less animal suffering compared to the blood samples harvested by heart puncture in a live full-term calf fetus.

The ECM is extremely complex particularly in solid tumours^29^ and the deposition of ECM by different cells indicate their contribution in modifying the TME^31^. Here, we have initiated the characterization of the ECM formed in our 3D PCL-based cultures by investigating filamentous ECM proteins with adhesion regulatory potential, such as fibronectin, collagen I, and laminin. A tumour is rich in various cytokines one of which is TGF-β1^32^. The source of TGF-β1 in tumours varies and includes both cancer cells and stromal cells^33^. Another cytokine found in the TME is IL-6, which plays important roles in cancer progression^21,34^. IL-6 is one of the key mediators of interaction between cancer cells, fibroblasts, and the TME^35^. There is substantial cross-talk between IL-6 and TGF-β1, which is associated with malignant features^36^. Here, we have investigated if TGF-β1 treatment affected the deposition of the studied proteins as well as on the level of IL-6 in the cell culture medium. TGF-β1 has been used at the concentration of 5 ng/ml in many in vitro studies. Chen and Thibeault^37^ investigated the response to TGF-β1 treatment of fibroblasts grown in 3D hyaluronan hydrogels.

Collagens are the most abundant fibrous glycoproteins in the normal and tumour ECM and many types of collagen has been identified^2^. Collagens of mainly types I, III, and IV have been demonstrated to show altered expression in the TME^8^. Fibroblasts are considered the major source of collagens found in the ECM. Since, we consider the PCL fibre network as a collagen mimicking structure, we were interested in the collagen deposition. Collagen I was found in the ECM of HDFs 3 days after seeding and the level then increased during the experimental period, implying that PCL per se did not totally hamper collagen deposition. Thus, the PCL fibre network mimicking the structure of collagen provides the possibility of 3D attachment and growth for the cells and collagen is formed by HDFs both in mono-culture and in co-culture with JIMT-1 cells.

The glycoprotein fibronectin constitutes a major portion of the ECM and mediates ECM assembly, ECM-cell interactions, and a wide variety of cellular activities such as adhesion, migration, growth, and differentiation^38^. Hielscher and colleagues^39^ have co-cultured dermal fibroblasts with breast cancer cells for establishment of fibronectin-rich 3D cell-derived decellularized matrix for angiogenesis studies. In line with these studies, the current work showed abundant deposition of fibronectin in our 3D PCL cultures and a slight increase in fibronectin deposition after TGF-β1 treatment was observed. TGF-β1 treatment of dermal fibroblasts has been shown to significantly increase the mRNA level and deposition of fibronectin^40^.

Laminins are versatile structural components abundantly found in mammalian basement membranes^41^. In tumours, high amounts of laminin are produced by both stromal cells and by epithelial cancer cells promoting tumour dissemination, migration, and invasion^42,43^. Here, we demonstrate increasing deposition of laminin during the 14 days experimental period in HDF mono-cultures and JIMT-1/HDF co-cultures. Further, laminin deposition was not significantly affected by TGF-β1 treatment. Fullár et al.^44^ found a significant increase in secreted laminin when cancer cells were grown in direct contact with fibroblasts in 2D cultures. Here, we do not have evidence of increased laminin deposition in the JIMT-1/HDF co-culture compared to the HDF mono-culture. However, it is not possible to totally compare the different culture conditions as we do not have exact quantitate data of cell numbers in the complex 3D system.

In a tumour, the ECM is constantly undergoing remodelling and the most common enzymes involved in this process are MMPs^11^. MMPs target a wide range of ECM proteins but each MMP mainly degrades a specific protein. We opted to determine the overall MMP activity in medium sampled from the cultures. In tissues, fibroblasts are responsible for the deposition of ECM proteins but they also secrete the ECM degrading MMPs^45^. Previous studies suggest that TGF-β1 treatment promoted the activities of MMPs in fibroblast cell extracts, however, they did not show activity in the medium of the cells^46^. Here, we show significant increase in MMP activity in TGF-β1-treated HDF cultures, implying an active role of MMPs in ECM remodelling. MMP mRNA expression has been found in many breast cancer cell lines, and MMP activity is related to the malignancy and aggressiveness of the cells^47,48^. MMP activity was found in JIMT-1 culture medium but it was only about one-fourth of the activity found in the medium of HDFs and TGF-β1 treatment resulted in the reduction of the MMP activity in the JIMT-1 medium. The MMP activity in the medium obtained from co-cultures is presumably derived from the HDFs. Presently, we have no explanation to why TGF-β1 treatment did not increase the MMP activity in the co-cultured cells as the co-culture also harbours HDFs. However, we did see an effect of TGF-β1 treatment on the 3D co-culture morphology where the cancer cells seem to spread out along the streaks of elongated HDFs in contrast to the tight clusters of cancer cells seen in co-cultures incubated in the absence of TGF-β1. This result agrees with the observation that fibroblasts alter the architecture and physical properties of the ECM through matrix remodelling and promote directional cancer cells migration^49^. TGF-β1 is furthermore known to stimulate epithelial to mesenchymal transition which is a feature of increased cell migration^50^.

Previous studies suggest that TGF-β1-induced phenotypic alterations of normal fibroblasts to myofibroblasts can be identified by their expression of the CAF markers alpha-smooth muscle actin (α-SMA) and fibroblast activation protein alpha (FAP-α)^51,52^. On the other hand, it has also been stated that neither α-SMA nor FAP-α are found in all CAFs and that morphology is the most reliable way to identify CAFs^53^. Several studies show that α-SMA expression is highly heterogenous among CAFs^54,55^. We did stain the HDFs in our cultures with both anti α-SMA and anti FAP-α antibodies but found no expression in either control or TGF-β1-treated cultures (not shown). However, we do see a marked change in morphology of TGF-β1-treated HDFs with much more distinct actin filaments compared to control HDFs, which has also been observed by others in CAFs^54^. Using RNA sequencing, it has recently been shown that CAFs in a tumour have different distinct phenotypes depending on their origin^53^ and to truly classify the HDFs in our 3D culture system, a further development is to use RNA sequencing methods.

EMT is a process in which cells obtain mesenchymal traits of invasion and motility^56^. TGF-β signalling has multiple roles in breast cancer metastasis stimulating EMT of both fibroblasts and cancer cells^16^. Much work has revealed the profound influence of fibroblasts on cancer progression and that TGF-β signalling is involved in the transformation of fibroblasts to migratory myofibroblasts which support the metastases of cancer cells^57^. Thus, the notion of targeting fibroblasts in tumours has been proposed as a novel anti-tumour therapy^58,59^. We believe the presented artificial 3D human tumour can be used for investigation of mechanisms of fibroblast activation by TGF-β1 as well as of how fibroblasts stimulate cancer cell migration and also for evaluation of fibroblast targeting compounds that may be further developed for clinical use.

IL-6 is a pro-inflammatory cytokine released in the breast TME by both cancer cells and stromal cells such as fibroblasts, tumour associated macrophages, and helper T cells^34^. Activation of IL-6 signalling pathways in cancer cells stimulates cell proliferation and migration. Previously, a dramatic and specific enhancement in IL-6 secretion as a potential mediator of cross-talk between tumour cells and CAFs has been identified in 3D tumouroids^35^. Here, we show that IL-6 in the medium of HDFs in 3D PCL-based cultures treated with TGF-β1 is increased significantly compared to control medium, which is in corroboration by results of others growing fibroblasts in collagen and Matrigel^60,61^. Activated fibroblasts have been shown to secrete more IL-6 and also MMPs contributing to increased matrix remodelling^62^. Thus, we assume the HDFs in 3D treated with TGF-β1 are activated. In our study, we detected approximately fivefold less IL-6 in the medium of JIMT-1 cells compared to the medium of HDFs and TGF-β1 treatment resulted in reduced IL-6 concentration in the medium of JIMT-1 cells. Interestingly, we found that the IL-6 concentration in the medium of co-cultures was 10 times higher than that in the medium of HDFs, however, this level was not significantly affected by TGF-β1 treatment. Presently, we have no explanation for this. The HDFs in co-cultures treated with TGF-β1 show similar morphology and increased actin expression as the HDFs in TGF-β1-treated mono-cultures. As already mentioned, the JIMT-1 cells have a spread-out metastatic behaviour in the TGF-β1-treated co-cultures compared to the control implying cellular effects related to IL-6 and MMP activity.

## Conclusions

We have constructed an artificial 3D human tumour which in many aspects resemble a human tumour in the body. We show that the growth pattern of cancer cells and fibroblasts in the artificial tumour resembles that of a tumour in vivo where the cancer cells form tight colonies surrounded by the fibroblasts. The ECM deposition in the artificial tumour also resembles that of a tumour in vivo. Importantly, we show that TGF-β1 treatment induces a metastatic like appearance of the artificial tumour with the cancer cells seemingly migrating along stretched out fibroblasts. We believe this model can be used to study various aspects of tumour development as well as for anti-cancer drug discovery.

## Data availability

The datasets used and/or analysed during the current study are available from the corresponding author on reasonable request.

## Supplementary Information

Supplementary Information

## Abbreviations

2D: Two-dimensional
3D: Three-dimensional
CAFs: Cancer associated fibroblasts
DHHS: Donor herd horse serum
ECM: Extracellular matrix
HDFs: Human dermal fibroblasts
PCL: Polycaprolactone
TGF-β1: Transforming growth factor-β1
TME: Tumour microenvironment
