# Supplementary material for: Identification of extracellular matrix proteins secreted by human dermal fibroblasts cultured in 3D electrospun scaffolds
Source: Sci Rep. 2021 Mar 23;11:6655. doi: 10.1038/s41598-021-85742-0 (PMC7988018; doi:10.1038/s41598-021-85742-0)
Supplement: Supplementary file 1 — Supplementary Information [file 41598_2021_85742_MOESM1_ESM.docx]

# **Identification of Extracellular Matrix Proteins Secreted by Human Dermal Fibroblasts Cultured in 3D Electrospun Scaffolds**

Atena Malakpour-Permlid, Irina Buzzi, Cecilia Hegardt, Fredrik Johansson, and Stina Oredsson

**Supplementary Material**


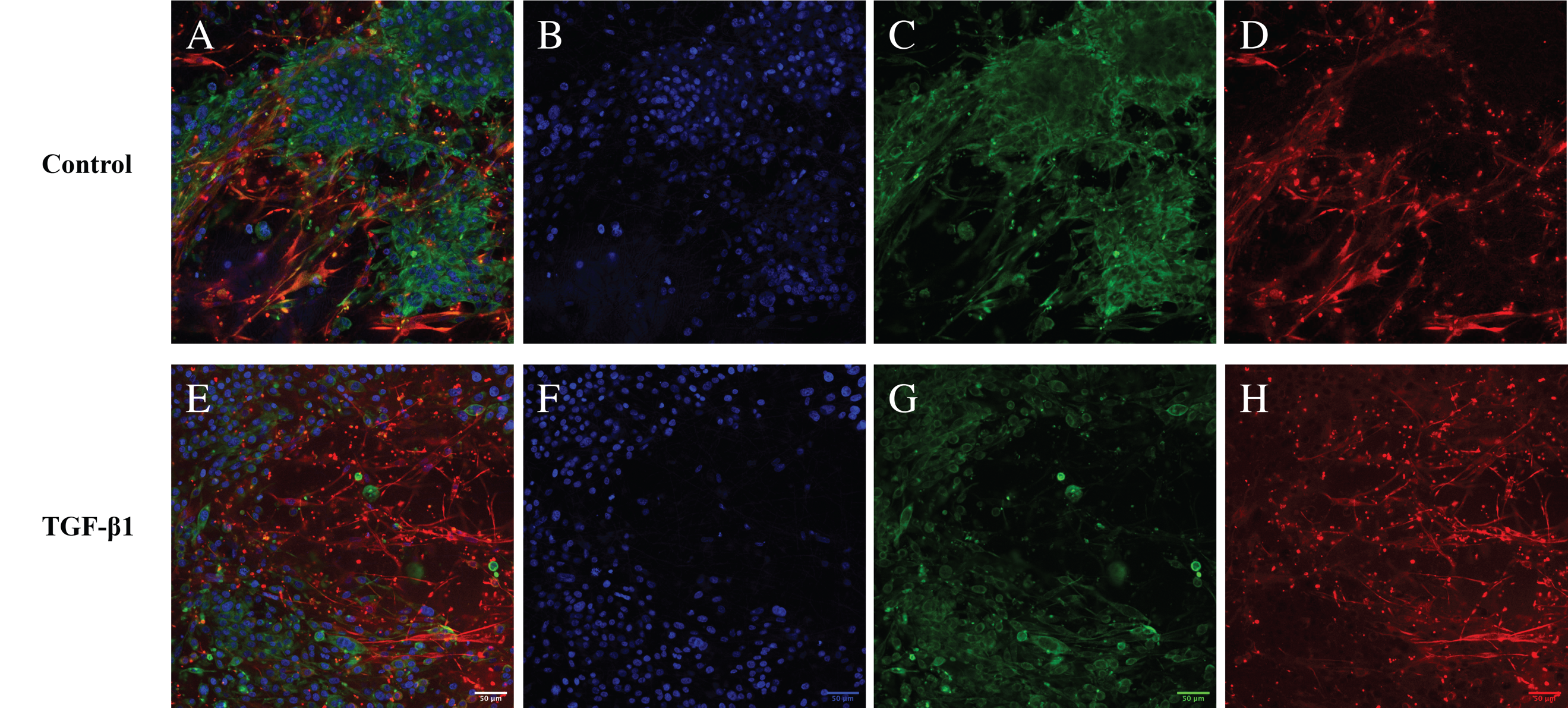


**Figure S1.** Intracellular localisation of IL-6 in 3D co-culture of JIMT-1 cells and HDFs showing low expression of IL-6 in HDFs. The cultures were incubated in the absence (control) or presence of 5 ng/ml TGF-β1. After 14 days of incubation, the cells were fixed with 3.7 % formaldehyde and stained to visualize IL-6 (green), vimentin (red), and cell nuclei (blue). Scale bar is 50 μm.
